# A comprehensive survey of polymorphisms conferring anti-malarial resistance in *Plasmodium falciparum* across Pakistan

**DOI:** 10.1186/1475-2875-12-300

**Published:** 2013-08-29

**Authors:** Aamer A Khattak, Meera Venkatesan, Christopher G Jacob, Elena M Artimovich, Muhammad F Nadeem, Farida Nighat, Francis Hombhanje, Toshihiro Mita, Salman A Malik, Christopher V Plowe

**Affiliations:** 1 Department of Biochemistry, Faculty of Biological Sciences, Quaid-i-Azam University, Islamabad, Pakistan; 2 Howard Hughes Medical Institute/Center for Vaccine Development, University of Maryland School of Medicine, Baltimore, MD, USA; 3 WorldWide Antimalarial Resistance Network Molecular Module, University of Maryland School of Medicine, Baltimore, MD, USA; 4 King Edward Medical University, Lahore, Pakistan; 5 Department of Biochemistry and Molecular Biology, University of Gujrat, Gujrat, Pakistan; 6 Islamic International Medical College, Rawalpindi, Pakistan; 7 Centre for Health Research, Divine Word University, Madang, Papua New Guinea; 8 Department of Molecular and Cellular Parasitology, Juntendo University, Tokyo, Japan

**Keywords:** *Plasmodium falciparum*, Malaria, Pakistan, Drug resistance, Sulphadoxine-pyrimethamine, Chloroquine, ACT, *pfcrt*, *pfmdr1*, *pfdhfr*, *pfdhps*

## Abstract

**Background:**

Few studies have been conducted in Pakistan to determine the efficacy of chloroquine and sulphadoxine-pyrimethamine (SP), which remain in use as treatment for *Plasmodium vivax* and in combination with artesunate to treat *Plasmodium falciparum*, respectively. In this study, samples from several sites across Pakistan were characterized to determine prevalence of molecular resistance markers in the *P. falciparum* chloroquine resistance transporter (*pfcrt*), multidrug resistance (*pfmdr1*), dihydrofolate reductase (*pfdhfr*) and dihydropteroate synthase **(***pfdhps*) genes and the origin of chloroquine-resistant *P. falciparum* parasites.

**Methods:**

Microscopy-confirmed malaria parasite-positive blood samples from 801 patients across the country were collected in 2011. Of these, 171 infections were identified by polymerase chain reaction (PCR) as *P. falciparum* and analysed by pyrosequencing for mutations conferring chloroquine resistance (*pfcrt* codons 72–76), multidrug resistance (*pfmdr1* N86Y, Y184F, S1034C, N1042D and D1246Y), pyrimethamine resistance (*pfdhfr*, C50R, N51I, C59R, S108N and I164L) and sulphadoxine resistance (*pfdhps,* S436A, A437G, K540E, A581G and A613T/S). *pfmdr1* gene copy number variation was determined by real-time PCR, and microsatellites flanking the *pfcrt* locus were typed to determine the origin of the chloroquine-resistant haplotype.

**Results:**

The *pfcrt* K76T mutation was found in all samples as part of the S72/V73/M74/N75/T76 (SVMNT) haplotype. Microsatellites flanking *pfcrt* showed high similarity to the signature found in India and Papua New Guinea. *pfmdr1* N86Y was found in 20% of samples and all samples harboured a single copy of the *pfmdr*1 gene. The *pfdhfr* double mutation C59R + S108N was present in 87% of samples while the *pfdhfr* triple mutant (N51I + C59R + S108N) was not detected. *Pfdhps* A437G was found in 60% of samples. Pure *pfdhps* K540E was rare, at 4%, but mixed genotype 540 K/E was found in 77% of samples. Similarly, pure *pfdhps* A581G was found in 4% of the isolates while mixed 581A/G was found in 39% of samples.

**Conclusions:**

These results suggest an emerging problem with multidrug resistant *P. falciparum* in Pakistan. The chloroquine resistance genotype has reached complete fixation in the population, with a microsatellite pattern indicative of a selective sweep. Moreover, the prevalence of mutations in both *pfdhfr* and *pfdhps*, albeit without the presence of the *pfdhfr* triple mutant, indicates that continued monitoring is warranted to assess whether SP remains efficacious as a partner drug for artesunate for the treatment of *P. falciparum*.

## Background

In Pakistan, an estimated 500,000 episodes of malaria infection occur annually [1]. Although the majority of these cases is attributed to *Plasmodium vivax*, studies indicate that *Plasmodium falciparum* in Pakistan has been on the rise over the past few decades [2]. The proportion of malaria infections attributed to *P. falciparum* rose from 34 to 54% between 1987 and 1990 in north-west Pakistan [3], and the frequency of *P. falciparum* among microscopy-positive cases rose from 45% in 1995 to 68% in 2006 in the provinces of Balochistan and Sindh [4,5]. In 2010, out of a total of 240,591 reported malaria cases in Pakistan, 73,857 (31%) were *P. falciparum*[6].

The first-line treatment of uncomplicated malaria of undetermined species in Pakistan is chloroquine. Chloroquine-resistant *P. falciparum* was first reported in Pakistan in 1984 [3] and subsequent *in vivo* studies have confirmed that chloroquine is not an efficacious treatment for *P. falciparum* in Central Asia. A survey conducted in western Pakistan in Afghan settlements during 1994–1995 reported *in vivo* chloroquine resistance ranging from 18 to 62% [7]. Both standard and extended-dose courses of chloroquine resulted in high levels of treatment failure in clinical studies conducted in Afghani refugees in Pakistan [8]. Other clinical studies conducted in refugee communities throughout the country indicated that chloroquine resistance increased as much as 15% in exposed populations in a single year, rising five- to six-fold between 1982 and 1992 [2].

In response to the declining efficacy of chloroquine, the World Health Organization (WHO) recommended artesunate plus sulphadoxine-pyrimethamine (AS + SP) as the first-line choice of treatment for uncomplicated *P. falciparum* malaria in Pakistan [9]. However, a 56% treatment failure rate with SP monotherapy was reported in Balochistan province in a study conducted between 2001 and 2005 [10], indicating that SP may be compromised as an effective partner drug.

Molecular surveillance of drug resistance has been used extensively to monitor sensitivity to chloroquine and SP. A point mutation causing an amino acid change from K to T at codon 76 of the *P. falciparum* chloroquine resistance transporter gene (*pfcrt*) confers resistance to chloroquine *in vitro*[11,12] and is strongly associated with treatment failure *in vivo*[13]. The role of these mutations in chloroquine resistance has also been confirmed by transfection studies [11]. A small number of independent *pfcrt* resistant lineages has been found: two in South America, one in Papua New Guinea [14] which has also been found in Asia [15,16], one observed in the Philippines, and one in Southeast Asia which spread to Africa [14,17].

Mutations in the dihydropteroate synthase (*pfdhps*) and dihydrofolate reductase (*pfdhfr*) genes of *P. falciparum* confer resistance to SP. Point mutations at positions N51I, C59R, S108N and I164L in *pfdhfr* are associated with pyrimethamine resistance *in vitro*[18-20] and treatment failure *in vivo*[21-23]. Resistance to sulfas is conferred by mutations at codons A437G, K540E, A581G and A613T/S of the *pfdhps* gene, confirmed by *in vitro*[24-28] and *in vivo*[22-24] studies. Resistance to SP occurs in a step-wise fashion, with an increasing number of mutations in *pfdhfr* and *pfdhps* contributing to an increased risk of treatment failure [29]. Infections carrying the *pfdhfr* triple mutant (S108N + N51I + C59R) are significantly more likely to fail SP treatment than infections with fewer *pfdhfr* mutations [30], and the *pfdhfr/pfdhps* quintuple mutant (*pfdhfr* triple mutant S108N + N51I + C59R plus the *pfdhps* double mutant A437G + K540E) is highly predictive of clinical failure in Africa [23,29,31].

Elevated copy number of the *pfmdr*1 gene is associated with susceptibility of *P. falciparum* to artemisinin, mefloquine, and lumefantrine [32-34], and has been used as a surveillance tool for artesunate-mefloquine resistance in Southeast Asia [33,35]. Mutations in the *pfmdr1* gene leading to the substitution of amino acids including N86Y, Y184F, S1034C, N1042D, and D1246Y also modulate *in vitro* susceptibility to a number of drugs [36], although their relevance in conferring clinical resistance remains uncertain.

In Pakistan, previous molecular surveys of drug resistance have focused on one or two sites in a small number of geographic regions. In these surveys, the most recent of which was conducted in 2007, high levels of *pfcrt* 76T have been reported [1,15,37], and multiple mutations in the *pfdhfr* and *pfdhps* genes have been found [1,37,38]. In this study, the prevalence of molecular markers of anti-malarial resistance was investigated in a comprehensive survey over 14 sites in four provinces of Pakistan and in the capital city of Islamabad. Collected in 2011, these samples provide information on the current distribution of drug-resistant genotypes in the *pfcrt*, *pfmdr1*, *pfdhfr* and *pfdhps* genes five years after the implementation of artesunate + SP as the first-line treatment for uncomplicated *P. falciparum.*

## Methods

### Study sites and ethics

**Figure 1:**
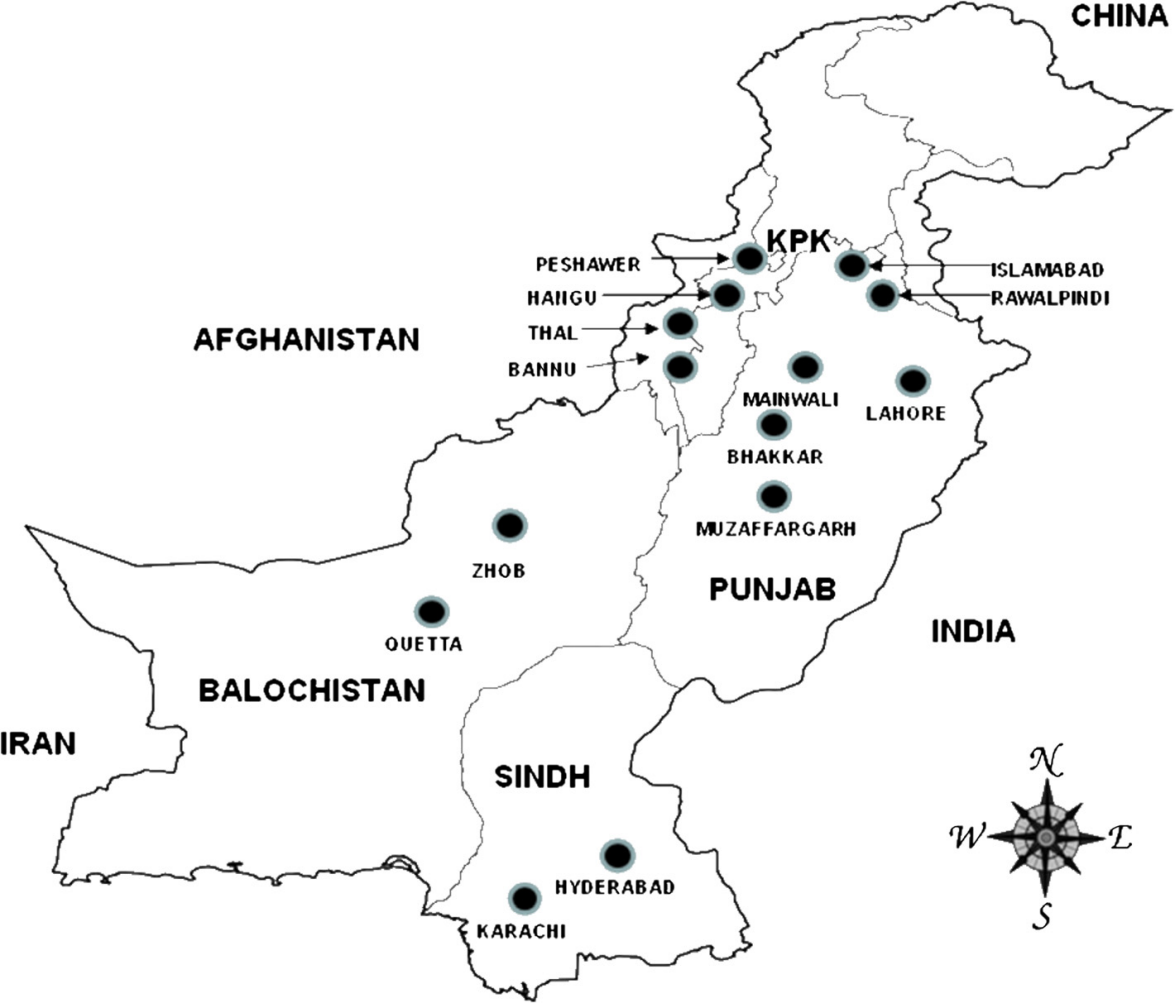
2011 collection sites with malaria-positive isolates: 14 cities in four provinces and the capital of Pakistan.

Government and private hospitals in 25 cities from four provinces with the highest burden of malaria representing all four provinces (Khyber Pakhtunkhwa province, Sindh province, Balochistan province and Punjab province) and a hospital in the capital city, Islamabad, were invited to participate in the molecular survey in 2011. Although the Federally Administered Tribal Areas have among the highest burdens of malaria in the country, they were excluded from this study because political instability and violence [39] make it difficult to establish sample collection. Of the facilities that were contacted, hospitals in 14 cities shared samples and were included in this study (Figure 1). Symptomatic patients of all age groups were enrolled and 3 mL of venous blood was drawn from each patient. Consent was obtained from patients or from children’s parents or guardians. Information on patient age and gender was also collected. The study was approved by the institutional review board of Quaid-i-Azam University, Islamabad, Pakistan.

### Microscopy and sample collection

Thick and thin blood films of patients with suspected malaria were stained with 10% Giemsa solution and examined at 1,000 × magnification under oil immersion by a laboratory technician or technologist trained in malaria diagnosis according to WHO guidelines [40]. Approximately 50 μl of each blood sample from 801 malaria microscopy-positive and 30 microscopy-negative samples was applied to Whatman 3MM filter paper. Filter papers were air-dried overnight and stored at room temperature in individual plastic bags with desiccant.

### DNA extraction and speciation

Molecular analysis was conducted at the Howard Hughes Medical Institute/University of Maryland School of Medicine in Baltimore, MD, USA. DNA was extracted from the blood-impregnated filter papers using the QIAmp 96 DNA kit (Qiagen, Valencia, CA, USA). *Plasmodium* species (*P. vivax* and *P. falciparum*) were confirmed by nested PCR amplification of the small subunit ribosomal ribonucleic acid (ssrRNA) genes by using the primers and thermal cycler conditions as previously described [41]. PCR products were visualized by 2–2.5% agarose gel electrophoresis using the Bio-Rad gel doc system. Extracted DNA was stored at −20°C for further analysis.

### Pyrosequencing of *pfdhfr, pfdhps, pfcrt*, and *pfmdr1* codons

PCR for pyrosequencing of *pfdhfr* codons C50R, N51I, C59R, S108N and I164L and *pfdhps* codons S436A, A437G, K540E, A581G and A613T/S was conducted using previously described cycling conditions [42] with a few modifications. Nested PCR reactions were prepared in 25 μl PCR reaction volume, contained 1 μl DNA template, 1× PCR buffer (Qiagen, Valencia, CA, USA), 0.2 mM dNTPs (Invitrogen) and 0.05 units HotStar Taq DNA polymerase (Qiagen, Valencia, CA, USA). The substitution of HotStar Taq required an initial incubation step of at 95°C for 15 min for all PCR reactions. PCR was performed busing a BioRad tetrad Thermal cycler (Bio-Rad, Hercules, CA). PCR primers and cycling conditions for pyrosequencing of *pfcrt* codons 72–76 and *pfmdr1* codons N86Y, Y184F, S1034C, N1042D and D1246Y are described on the investigator’s website [43].

Pyrosequencing for all genes was performed using PyroMark® Q96 MD pyrosequencer, using the protocol previously described [42]. Between 2 and 7 μl of secondary PCR product or sequence-specific amplified positive control DNA provided by Malaria Research Reagent Resource (MR4) (Manassas*,* Virginia, USA*)* was added to each pyrosequencing reaction, depending on the on the PCR product yield. Single nucleotide polymorphisms (SNPs) were called using PyroMark® Q96 MD pyrosequencing software version 1.2 (Qiagen) in allele quantification mode (AQ) for all SNPs except for *pfcrt* 72*–*76 and *pfmdr1* N86Y, which were analysed in sequence analysis mode (SQA). Single peak signals of at least 30 RLU (relative luminescence units) were considered suitable for allele quantification. Pyrosequencing was repeated with an adjusted amount of PCR product for samples failing to produce a SNP call because of too much DNA (saturating the pyrogram) or insufficient DNA (producing a weak signal).

SNP calls obtained by pyrosequencing were adjusted using standard curve equations derived from pyrosequencing of mixtures of control DNA strains with known proportions for each allele. To account for machine error, the lower cutoff for inclusion of minor alleles in a mixed infection was set at 10%. *pfcr*t K76T and *pfmdr*1 N86Y could not be absolutely quantified using pyrosequencing so a standard curve could not be applied. The minor allele frequency cutoff for these two codons was set at 20% to account for both machine error and lack of adjustment by standard curves.

Isolates were considered to be ‘pure’ mutants if only the resistance-conferring codon was observed, and ‘mixed’ if both the wild-type and mutant allele were detected. Similarly, haplotypes (combinations of polymorphisms) were considered ‘pure’ mutants if wild-type alleles were not detected in any of the SNP positions, and mixed if both wild-type and mutant alleles were detected in at least one SNP position.

### *pfmdr1* copy number variation

*pfmdr1* copy number was assessed by TaqMan real-time PCR (ABI sequence detector 7700; Applied Biosystems, Warrington, UK) using previously described primers, probes, and reaction conditions [33]. Every qPCR run contained genomic DNA extracted from *P. falciparum* strains of 3D7, Dd2 and K1 with *pfmdr1* copy numbers, of 1, 2, and 3 respectively. Each sample in this assay was run in triplicate. Assays were repeated if one of the following three results was obtained: ∆∆Ct spread > 1.5; Ct values > 35; or copy number estimates between 1.3 and 1.6 [33].

### Microsatellite genotyping

Six microsatellites, located 2.8, 4.3, 10.8, and 29.3 kb upstream of *pfcrt* and 0.6 and 10.4 downstream of *pfcrt* on chromosome 7, were used for genotyping. These microsatellites were amplified using heminested PCR and fluorescently labeled primers [44]. Specifics of PCR reactions, thermal cycling conditions, and fragment size calling were carried out as described by Laufer *et al.*[45]. In addition to samples collected in this study, four *P. falciparum* DNA samples collected between 2005 and 2007 in a previous study in Pakistan [15] and four collected in Dagua Province in Papua New Guinea in 2010 were genotyped at all six microsatellite loci to assess shared ancestry of the *pfcrt* locus.

## Results

A total of 801 microscopy-positive samples was collected from 14 sites. Of these isolates, 128 samples were identified by PCR as *P. falciparum* and an additional 43 samples contained both *P. falciparum* and *P. vivax* infections (Table 1) for a total of 171 samples containing *P. falciparum*. Sixty three percent (108 samples) were collected from male subjects and 37% (63 samples) were collected from females. Subject age ranged from three months to 75 years, with a median of 24 years.

**Table 1:** ***Plasmodium falciparum *samples collected from 14 sites in Pakistan**

**Province**	**City**	**PCR-confirmed*P. falciparum*(N)**
**Khyber Pakhtunkhwa**	Bannu	39
	Hangu	19
	Peshawar	4
	Thal	29
**Punjab**	Bhakkar	1
	Lahore	1
	Mianwali	28
	Muzaffarghar	13
	Rawalpindi	0
**Balochistan**	Quetta	26
	Zhob	7
**Sindh**	Hyderabad	0
	Karachi	2
**Capital**	Islamabad	2
**Total**		171

Pyrosequencing of *pfdhfr* and *pfdhps* genes harbouring mutations conferring drug resistance was carried out in 171 PCR-positive *P. falciparum* isolates. The *pfdhfr* triple mutant N51I + C59R + S108N was detected in 7 samples, all carrying a mixture of wild-type and mutant alleles (Table 2). The *pfdhfr* double mutant C59R + S108N was found in 148 samples (87%) (Table 2). The *pfdhps* mutant codon A437G was observed in 61 samples (51.5%), with few mixed infections. *pfdhps* K540E was found in six samples, and mixed K/E infections were found in 131 samples (77%). *pfdhps* A581G was observed in five samples while 66 samples (39%) had the mixed A/G genotype. *pfdhfr*/*pfdhps* C59R + S108N + A437G was found in 88 isolates (51%).

**Table 2:** **Number (N) and prevalence (%) of pure/mixed *pfcrt*, *pfmdr1*, *pfdhfr *and *pfdhps *mutant alleles conferring resistance to chloroquine and sulphadoxine-pyrimethamine in *Plasmodium falciparum *isolates from Pakistan**

		**Pure**	**Mixed**
**Gene**	**Allele or haplotype**	**N (%)**	**N (%)**
***pfcrt***	SVMNT/ K76T	171 (100)	0 (0)
***pfmdr1***	N86Y	6 (4)	28 (16)
	Y184F	0 (0)	43 (25)
	S1034C	0 (0)	0 (0)
	N1042D	0 (0)	165 (96)
	D1246Y	0 (0)	0 (0)
***pfdhfr***	*pfdhfr* triple (N51I + C59R + S108N)	0 (0)	7 (4)
	*pfdhfr* double (N51I + S108N)	11 (6)	3 (2)
	*pfdhfr* double (C59R + S108N)	148 (87)	9 (5)
***pfdhps***	*pfdhps* double (A437G + K540E)	4 (2)	77 (45)
	*pfdhps* A437G	103 (60)	3 (2)
	*pfdhps* K540E	6 (4)	131 (77)
	*pfdhps* A581G	5 (3)	66 (39)
***pfdhfr + pfdhps***	*pfdhfr* + *pfdhps* (C59R + S108N + A437G)	88 (51)	6 (4)

The distribution of common *pfdhfr* and *pfdhps* haplotypes (combinations of polymorphisms) by region is shown in Table 3. Prevalence of *pfdhfr* double mutant C59R + S108N ranged from 72% in Punjab (in 31 of 43 samples) to 95% in Khyber Pakhtunkhwa (in 87 of 91 samples). *pfdhfr* C59R + S108N + *pfdhps* A437G was present in 44-58% of samples collected in Punjab, Khyber Pakhtunkhwa, and Balochistan (sample sizes of 43, 91, and 33, respectively). Only two *P. falciparum* samples each was collected from Islamabad and Sindh, and all four carried *pfdhfr* C59R + S108N + *pfdhps* A437G.

**Table 3:** **Number and prevalence of SP resistance-associated haplotypes in *pfdhfr *and *pfdhps *in Pakistan, by province**

		**Number and prevalence (%) of*pfdhfr*,*pfdhps*, and*pfdhfr*** **+** ***pfdhps*alleles**							
		***pfdhfr*N51I + C59R + S108N**	***pfdhfr*N51I + S108N**	***pfdhfr*C59R + S108N**		***pfdhps*A437G + K540E**		***pfdhfr*C59R + S108N +** ***pfdhps*A437G**	
	**n**	Mixed*	Pure**	**Pure**	**Mixed**	**Pure**	**Mixed**	**Pure**	**Mixed**
Balochistan	33	4 (12)	1 (3)	26 (79)	1 (3)	2 (6)	16 (49)	19 (58)	0
KPK***	91	0	2 (2)	87 (95)	1 (1)	1 (1)	32 (35)	46 (51)	1 (1)
Islamabad	2	0	0	2 (100)	0	0	2 (100)	2 (1)	0
Punjab	43	3 (7)	8 (19)	31 (72)	1 (2)	1 (2)	25 (58)	19 (44)	5 (12)
Sindh	2	0	0	2 (100)	0	0	2 (100)	2 (100)	0
Total	171	7 (4)	11 (6)	148 (87)	3 (2)	4 (2)	77 (45)	88 (52)	6 (4)

*Mixed pfdhfr N51I + S108N not detected.

**Pure pfdhfr triple mutant not detected.

***KPK: Khyber Pakhtunkhwa.

All 171 samples analysed were amplified successfully for *pfcrt*. DNA pyrosequencing of the secondary PCR products from these samples confirmed that all of the amplified samples carried codon K76T (nucleotide sequence ACA). All samples were of the SVMNT haplotype for *pfcrt* 72–76 (Table 2).

*Pfmdr1* N86Y pure mutants were identified in six (4%) of the tested isolates, 28 (16%) were identified as mixed N/Y infections (Table 2). Forty-three (25%) isolates carried the mixed Y/F codon at position Y184F of the *pfmdr1* gene and the remaining samples carried the wild-type Y codon. One hundred and sixty-five (96%) isolates carried the mixed N/D polymorphism at position N1042D. All samples were wild-type for codons S1034C and D1246Y. Real time PCR to determine copy number variation in *pfmdr1* gene revealed that all isolates harboured a single copy of the *pfmdr1* gene.

All six microsatellite loci amplified successfully in 160 of 171 *P.falciparum* samples. Three isolates were mixed-strain infections with multiple alleles at each locus and were excluded from further analysis. Three haplotypes were observed in the remaining sample set of 157: the predominant haplotype, found in 137 samples, and two haplotypes in 12 and eight samples, respectively, each differing from the predominant haplotype at one microsatellite locus (Table 4). These *pfcrt* flanking microsatellite haplotypes were identical or nearly identical (differing in 1 locus) to those found in isolates with the SVMNT haplotype from Pakistan [15] and Papua New Guinea (Table 4).

**Table 4:** **Microsatellite haplotypes of *Plasmodium falciparum *parasites in the region flanking the *pfcrt *locus on chromosome 7**

			Microsatellites flanking pfcrt locus*					
**Country of origin**	**Number or strain**	***pfcrt*haplotype**	**−29**	**−11**	**−4**	**−3**	**+1**	**+10**
Pakistan (current study)	137	SVMNT	**147**	**168**	**230**	**178**	**151**	**203**
	12	SVMNT	-	-	-	182	-	-
	8	SVMNT	-	-	-	-	-	197
Pakistan^**^	4	SVMNT	-	-	-	-	-	-
Papua New Guinea	2	SVMNT	-	-	-	-	-	-
	2	SVMNT	149	-	-	-	-	-
Papua New Guinea	D10	CVMNK	-	170	-	166	-	193
Thailand	K1	CVIET	-	-	228	-	149	200
Papua New Guinea	FC27	CVIET	-	170	-	166	149	193
Indochina	Dd2	CVIET	-	176	228	-	149	200
Vietnam	V1S	CVIET	-	176	228	-	149	200
Indochina	W2	CVIET	-	176	228	-	149	200
Africa	3D7	CVMNK	149	180	-	180	149	-
Brazil	7G8	CVMNT	149	174	-	-	157	200
Honduras	HB3	CVMNK	149	182	-	184	147	193
Sierra Leone	D6	CVMNK	145	186	232	186	149	200

*Numbers indicate distance in kilobases upstream (−) and downstream (+) of the *pfcrt* locus.

**Reference [14].

## Discussion

In this study, polymorphisms in the *pfdhfr*, *pfdhps*, *pfcrt* and *pfmdr1* genes were investigated in *P. falciparum* samples collected throughout Pakistan to determine the current extent of resistance to chloroquine and antifolates, both of which are still used as part of first-line treatment of malaria in Pakistan.

The complete saturation of *pfcrt* K76T in all parts of the country indicates that chloroquine-resistant *P. falciparum* is fixed in Pakistan. These findings corroborate previous molecular studies reporting near fixation of chloroquine resistance [15,37] and clinical studies reporting high treatment failure of chloroquine [2,7,8]. Chloroquine resistance has also been documented in several neighbouring countries, including Iran, India, China and Afghanistan [46-50]. Although chloroquine is recommended for *P. vivax* infections only, *P. falciparum* infections are often exposed to this treatment as well. Presumptive diagnosis based on clinical grounds, improper diagnosis, lack of diagnostic facilities, and empirical treatment are common practices in resource-limited countries such as Pakistan [51,52], and these factors likely result in the continued mistreatment of *P. falciparum* infections with chloroquine. Such ineffective treatment of chloroquine-resistant *P. falciparum* with chloroquine can lead to increased morbidity and mortality if infections do not respond to the drug. Use of chloroquine to treat *P. falciparum* has also been postulated as contributing to the rapid growth of *falciparum* malaria in Pakistan [2], as infections may not clear and continue to circulate in the population unchecked.

Typing of microsatellites surrounding the *pfcrt* locus indicates that a selective sweep of chloroquine-resistant *P. falciparum* occurred in Pakistan, and that the SVMNT allele in Pakistan shares its ancestry with parasites in Papua New Guinea. Previous studies have shown that the SVMNT allele of the *pfcrt* locus appears to have swept through Papua New Guinea [14], multiple parts of India [16,46] and a small number of sites Pakistan [15]. Taken together with the findings in this study, these results suggest that the spread of SVMNT may have been a single sweep event through a large region encompassing parts of the South Pacific and South Asia. The possibility that such a large sweep could occur again in the context of artemisinin resistance in this region should be carefully considered.

The current first-line treatment for confirmed *P. falciparum* malaria in Pakistan is artesunate + SP. The *pfdhfr* C59R + S108N double mutant was the most frequent pyrimethamine-resistant haplotype found, as has been shown in previous studies in Pakistan [1,37], India [47,48], Iran [38,49], and Sri Lanka [50]. The majority of samples also carried a mutation at position A437G and a number of samples harboured mixed infections at highly resistant codons K540E and A581G in the *pfdhps* gene. Although the clinically significant *pfdhfr* triple mutation was rare (and present only in mixed wild-type/mutant infections) and *pfdhfr/pfdhps* quintuple mutants were not detected, the presence of multilocus resistance haplotypes and the presence of highly resistant sulphadoxine alleles indicate that clinically relevant SP resistance could arise or be already present. A recent clinical trial in India reported artesunate + SP efficacy dipping to near the WHO-established efficacy threshold of 90%, likely attributable to the presence of multiple *pfdhfr* and *pfdhps* mutations [53].

All samples harboured a single copy of the *pfmdr1* gene, suggesting that artesunate, lumefantrine, and mefloquine are likely to have high efficacy in Pakistan, and that the latter two could be considered as alternate ACT partner drugs if the prevalence of SP resistance markers rises. Presence of point mutations at position N86Y, Y184F and N1042D of *pfmdr1* indicate that continued molecular monitoring of this gene may provide informative insights on its association with artemisinin and partner drugs in this region.

## Conclusions

The fixation of chloroquine resistance marker *pfcrt* K76T in Pakistan suggests that, in regions with known high levels of *P. falciparum*, improved species diagnosis and appropriate treatment with ACT are critical to ensure that malaria patients receive effective care. The presence of multiple resistance alleles in *pfdhfr* and *pfdhps* demonstrates the utility of molecular surveillance to monitor the efficacy of artesunate + SP in Pakistan. Based on these findings, molecular and therapeutic efficacy studies to assess the continued efficacy of artesunate + SP are warranted.

## Competing interests

The authors declare that they have no competing interests.

## Authors’ contributions

AAK designed study, carried out the laboratory experiments, and performed data analysis. AAK and MV drafted the manuscript. CGJ, MA and EMA contributed to laboratory experiments and data analysis. MFN and FN participated in sample data collection and microscopy. FH and TM contributed to sample collection and contributed advice for microsatellite data analysis. MV, SAM and CVP provided guidance and coordination for study design, genotyping, and data analyses, and edited and revised the manuscript. All authors read and approved the final manuscript.
